# Conventional and recent advances in gravity separation technologies for coal cleaning: A systematic and critical review

**DOI:** 10.1016/j.heliyon.2023.e13083

**Published:** 2023-01-21

**Authors:** Theerayut Phengsaart, Palot Srichonphaisan, Chinawich Kertbundit, Natatsawas Soonthornwiphat, Somthida Sinthugoot, Nutthakarn Phumkokrux, Onchanok Juntarasakul, Kreangkrai Maneeintr, Apisit Numprasanthai, Ilhwan Park, Carlito Baltazar Tabelin, Naoki Hiroyoshi, Mayumi Ito

**Affiliations:** a Department of Mining and Petroleum Engineering, Faculty of Engineering, Chulalongkorn University, Bangkok 10330, Thailand; b Division of Sustainable Resources Engineering, Faculty of Engineering, Hokkaido University, Sapporo 060-8628, Japan; c Department of Groundwater Resources, Ministry of Natural Resources and Environment, Bangkok 10900, Thailand; d Department of Geography, Faculty of Education, Ramkhamhaeng University, Bangkok 10240, Thailand; e Department of Earth Sciences, Faculty of Science, Kasetsart University, Bangkok 10900, Thailand; f Department of Materials and Resources Engineering Technology, College of Engineering and Technology, Mindanao State University-Iligan Institute of Technology, Iligan City 9200, Philippines

**Keywords:** Coal, Coal cleaning, Gravity separation, Separation technology

## Abstract

“Affordable and clean energy” is enshrined in the UN Sustainable Development Goals (SDGs; #7) because of its importance in supporting the sustainable development of society. As an energy source, coal is widely used because it is abundant and its utilization for electricity and heat generation do not require complex infrastructures and technologies, which makes it ideal for the energy needs of low-income and developing countries. Coal is also essential in steel making (as coke) and cement production and will continue to be on high demand for the foreseeable future. However, coal is naturally found with impurities or gangue minerals like pyrite and quartz that could create by-products (e.g., ash) and various pollutants (e.g., CO_2_, NO_X_, SO_X_). To reduce the environmental impacts of coal during combustion, coal cleaning—a kind of pre-combustion clean coal technology—is essential. Gravity separation, a technique that separates particles based on their differences in density, is widely used in coal cleaning due to the simplicity of its operation, low cost, and high efficiency. In this paper, recent studies (from 2011 to 2020) related to gravity separation for coal cleaning were systematically reviewed using the Preferred Reporting Items for Systematic Reviews and Meta-Analyses (PRISMA) guidelines. A total of 1864 articles were screened after removing duplicates, and after a thorough evaluation 189 articles were reviewed and summarized. Among of conventional separation techniques, dense medium separator (DMS), particularly dense medium cyclone (DMC), is the most popular technologies studied, which could be attributed to the growing challenges of cleaning/processing fine coal-bearing materials. In recent years, most of works focused on the development of dry-type gravity technologies for coal cleaning. Finally, gravity separation challenges and future applications to address problems in environmental pollution and mitigation, waste recycling and reprocessing, circular economy, and mineral processing are discussed.

## 1 Introduction

**Fig. 1:**
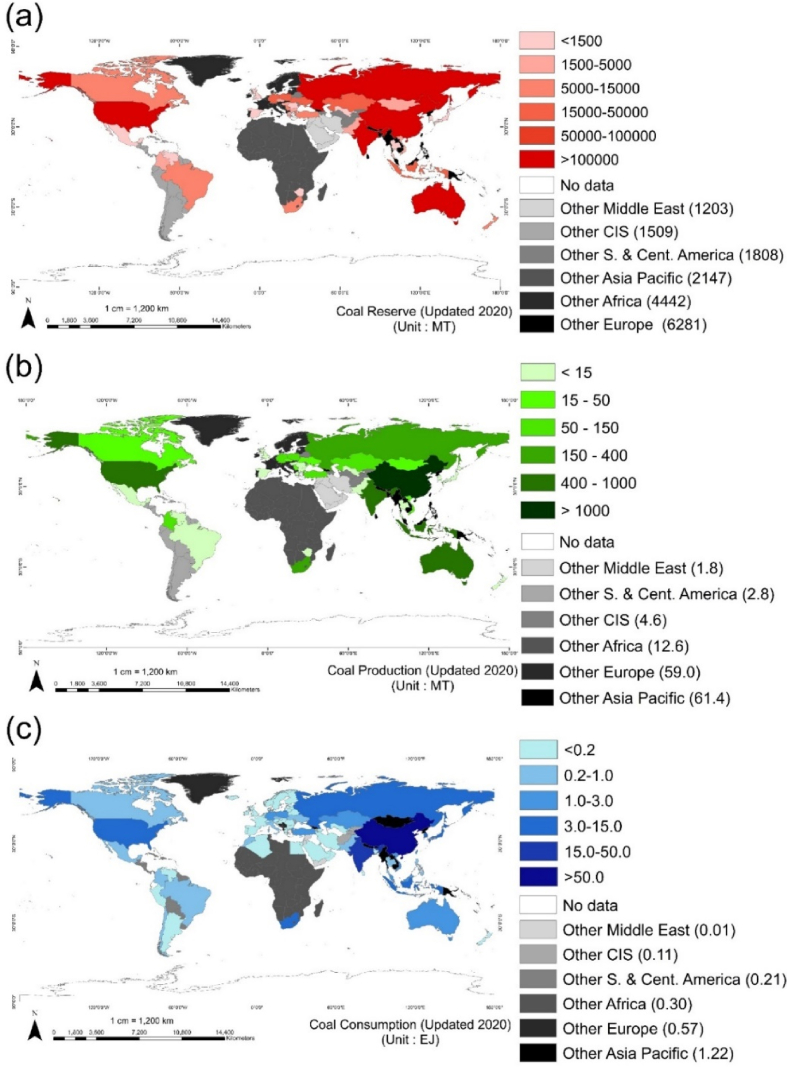
Choropleth maps of coal (a) reserve, (b) production, and consumption of coal (Updated 2020) [1]. Note: CIS = Commonwealth of Independent States.

Coal, a solid carbon-rich combustible material, is one of the most abundant and important fossil fuel used worldwide. It is essential in the electricity and heat generation sectors, production of cement and paper as well as in smelting of iron ore to steel (as coke). In 2020, the total proven coal reserves are estimated to last for 139 years at the current rate of consumption, which is longer when compared to the lifespans of crude oil and natural gas reserves (∼50 years each) [1]. As shown in Fig. 1 and Table 1, ∼75% of coal is found in only 5 countries—USA, Russia, Australia, China, and India—while the biggest coal consumers are China (54%), India (18%), USA (6%), Japan (3%), and South Africa (2.3%). In terms of production, China tops the list supplying about 50% of coal demand worldwide. Other important players in the global coal trade include India, Indonesia, USA, and Australia.

**Table 1:** Reserves, production, and consumption of coal [1].

Rank	Reserves			Production			Consumption		
	Country	Amount (MT)	%	Country	Amount (MT)	%	Country	Amount (MT)	%
1	USA	248,941	23.18	China	3902.0	50.40	China	82.41	54.43
2	Russia	162,166	15.10	India	756.5	9.77	India	17.54	11.58
3	Australia	150,227	13.99	Indonesia	562.5	7.27	USA	9.20	6.08
4	China	143,197	13.33	USA	484.7	6.26	Japan	4.57	3.02
5	India	111,052	10.34	Australia	476.7	6.16	South Africa	3.48	2.30
Others		258,525	24.06		1559.2	20.14		34.22	22.59
Total		1,074,108	100		7741.6	100		151.42	100

Coals are commonly classified into two types based on their final end use: (i) thermal coal, sometimes called steam coal or brown coal, for electricity and heat generation, and (ii) metallurgical coal, sometimes called coking coal or black coal, for steelmaking. Another classification used for coals is coal rank—a measure of the degree of organic metamorphism or coalification—and is based on standards and guidelines provided by the American Society for Testing Materials (ASTM). The ASTM D388 standard (Table 2) classify coal into four major types—anthracite, bituminous, subbituminous, and lignite—based on parameters obtained by various prescribed tests, which include calorific or heating value, volatile matter, moisture, ash, and fixed carbon. Each major type in this classification is further divided into sub-categories based on the physical, chemical, and technical properties of the coal [2].

**Table 2:** Classification of coal rank by ASTM D388 [2].

Class	Group	Fixed carbon limits* [%]	Volatile matter limits* [%]	Gross calorific value limits**		Agglomerating character
				[Btu/lb]	[MJ/kg]	
Anthracite	Meta-anthracite	≥98	≤2	–	–	Non-agglomerating
	Anthracite	92–98	2–8	–	–	
	Semi-anthracite	86–92	8–14	–	–	
Bituminous	Low volatile bituminous coal	78–86	14–22	–	–	Commonly agglomerating
	Medium volatile bituminous coal	69–78	22–31	–	–	
	High volatile A bituminous	≤69	≥31	≥14,000	≥32.6	
	High volatile B bituminous coal	–	–	13,000–14,000	30.2–32.6	
	High volatile C bituminous coal	–	–	11,500–13,000	26.7–30.2	
Subbituminous	Subbituminous A	–	–	10,500–11,500	24.4–26.7	Non-agglomerating
	Subbituminous B	–	–	9500–10,500	22.1–24.4	
	Subbituminous C	–	–	8300–9500	19.3–22.1	
Lignite	Lignite A	–	–	6300–8300	14.7–19.3	Non-agglomerating
	Lignite B	–	–	≤6300	≤14.7	

Note: * dry, mineral-matter-free basis.

** moist, mineral-matter-free basis.

The ratio between combustible organic matter and inorganic impurities of coal is also one of the variables in coal classification. Coal gangue is a significant residue of coal mining and cleaning, which accounts for approximately 10–15% of raw coal [3]. The first group of impurities in coal, which are also the most commonly encountered, are quartz (SiO_2_) and clay minerals like kaolinite (Al_2_Si_2_O_5_(OH)_4_), illite ((K,H_3_O)(Al,Mg,Fe)_2_(Si,Al)_4_O_10_ ((OH)_2_, (H_2_O))), and montmorillonite ((Na,Mg,Al)Si_4_O_10_(OH)_2_). The second group of impurities comprises carbonate minerals like calcite (CaCO_3_) and dolomite (Ca,Mg(CO_3_)_2_) while the third group includes oxides (e.g., hematite (Fe_2_O_3_)), sulfides (e.g., pyrite (FeS_2_), arsenopyrite (FeAsS), and sphalerite (ZnS)), phosphates (e.g., fluorapatite (Ca_5_(PO_4_)_3_F)), and silicates (e.g., muscovite (Kal_2_(Si_3_AlO_10_)(OH,F)_2_), feldspars (KalSi_3_O_8_–NaAlSi_3_O_8_ – CaAl_2_Si_2_O_8_), tourmaline (Al_6_B_3_Fe_3_H_10_NaO_31_Si_6_), and chlorite ((Mg,Fe)_3_(Si,Al)_4_O_10_(OH)_2_·(Mg,Fe)_3_(OH)_6_). Finally, the last group are environmentally regulated elements—arsenic (As), boron (B), cadmium (Cd), copper (Cu), manganese (Mn), mercury (Hg), selenium (Se), lead (Pb), and zinc (Zn)—enriched in coals because during coal formation, these elements are preferentially partitioned with carbonaceous materials [[4], [5], [6]], evaporite salts [3,7,8], and sulfide minerals like pyrite [[9], [10], [11], [12]].

**Fig. 2:**
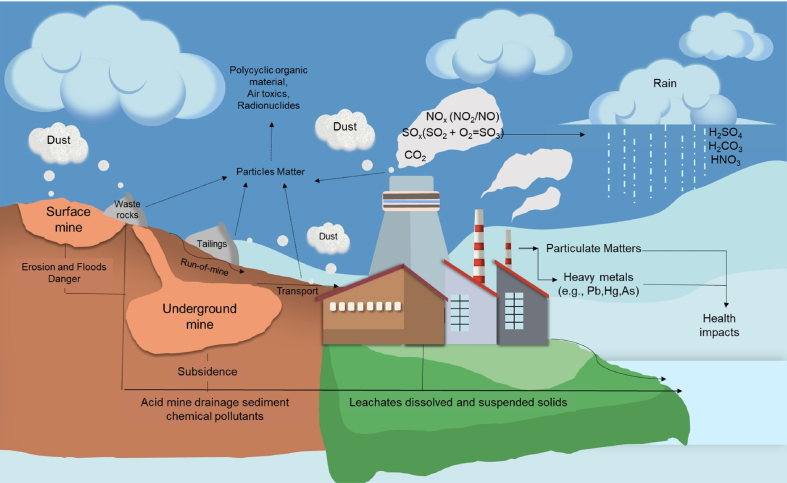
Environmental impacts through the life cycle of coal.

Fig. 2 illustrates how coal mining, handling, processing, utilization, and waste disposal affect the environment. The most notorious and widely reported impacts of coal utilization is related to the air. Coal combustion causes serious environmental issues like the generation of fly and bottom ashes containing soluble B and Se, release of particulate matter (PM), and emissions of hazardous pollutants like Hg, As, CO_2_, SO_x_, and NO_x_ to the air [[13], [14], [15]]. Boron and Se are essential micronutrients but toxic at high concentrations to animals and humans [16,17]. In contrast, Hg and As are both toxic even in minute concentrations and could cause diseases of the central nervous system and increase the risks of developing various types of cancers [[18], [19], [20]]. Meanwhile, NO_x_ are harmful gases because of their reactivity, CO_2_ is a well-known greenhouse gas that contributes to climate change, and SO_x_ easily dissolves in water vapor and droplets causing acid rain [21]. In cases where run-of-mine (ROM) coals are directly utilized without removing majority of the impurities, the high content of impurities is not only detrimental to boilers and economizers (e.g., corrosion) but also increases the emission of hazardous elements, particulates, and gases. In modern coal-fired power plants, the majority of these pollutants are removed from combustion gases through dust suppression and gas scrubbing technologies. Incombustible materials from gangue minerals becomes the ashes (fly and bottom ashes). In some countries, these ashes are disposed of in special landfills handling hazardous wastes. However, recently, both of fly and bottom ashes were utilized for many purposes in cement industries as well as agriculture [14,22].

Acid mine drainage (AMD) or acid rock drainage (ARD) is the most notorious environmental problem associated with coal mining, processing, and waste management that affect land and water. AMDs/ARDs—strongly acidic effluents polluted with hazardous and toxic metalloids and heavy metals—are formed when sulfide minerals like pyrite, sphalerite, and arsenopyrite ubiquitous in tailings/rejects, overburden/waste rocks, and mine workings are exposed to oxygen and water [[23], [24], [25], [26]]. The dissolution of sulfide minerals like pyrite could releases hazardous elements into the environment because this mineral is well-known to incorporate elements with similar chemical properties as Fe (e.g., Pb, Zn, Cd, Mn, and Cu) and S (e.g., As and Se) [[27], [28], [29], [30], [31], [32], [33]]. The AMD/ARD could be treated by neutralization reactions [[34], [35], [36]], sorption-precipitation [[37], [38], [39], [40]], galvanic interactions [[41], [42], [43]], microbial and electrochemically mediated processes like ferrous oxidation to ferric ions [[44], [45], [46]] and metal-organic complexation reactions [[47], [48], [49], [50]].

Although renewable energy and clean storage technologies are being championed by developed and high-income countries to replace fossil-fuel based technologies and combat climate change (UN Sustainable Development Goals (SDG) #13 “Climate action”) [51,52], this strategy is not applicable to low-income and developing countries considering the costs of these clean technology, including the vast infrastructure they require to be effective. An alternative is the development and deployment of clean coal technologies to mitigate coal-related environmental problems, turn coal into a cheap, clean and sustainable energy resources, and contribute to the UN-SDG #7 “Affordable and clean energy”. Moreover, there is still rare alternative for coal in steelmaking and cement manufacturing, so clean coal technologies can help limit the environmental impacts of these industries. Clean coal technologies can be divided into three categories: (i) pre-combustion technologies (e.g., coal cleaning, coal briquetting, coal liquid mixture, coal liquefaction, coal gasification): that pre-treats coal using physical and/or chemical methods to remove undesirable substances and impurities, such as dust, ash, rocks, and pyritic sulfur [[53], [54], [55], [56], [57]], (ii) in-combustion technologies (e.g., advanced pulverized coal combustion (APCC), fluidized bed combustion (FBC), integrated gasification combined cycle (IGCC), and low NO_X_ burners), which control and reduce SO_x_, NO_x_, CO_2_, and pollutant emissions during coal combustion, and (iii) post-combustion technologies (e.g., fabric filter, electrostatic precipitator (ESP), scrubbers, flue gas desulfurizer (FGD), selective catalytic reduction (SCR), solid waste utilization, and carbon dioxide capture, utilization, and storage (CCUS)) that remove and treat CO_2_, SO_2_, SO_3_, NO_x_, Hg, and dust after coal combustion [[58], [59], [60], [61], [62], [63], [64], [65]].

**Fig. 3:**
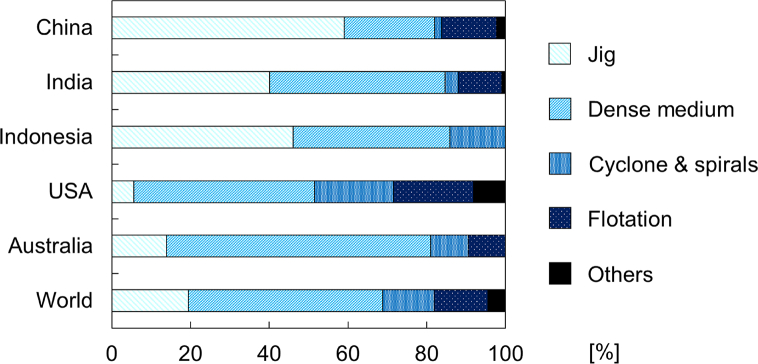
Usage of coal cleaning methods in top 5 coal production countries and worldwide [67].

Among the various clean coal technology options, coal cleaning—a type of pre-combustion technology—is one of the most promising and sustainable strategies because it reduces the environmental impacts of coal by removing majority of impurities prior to combustion [66]. Separation technologies to remove gangue minerals and impurities in coal are based on mineral processing technologies and a summary of the largest mines in top 5 countries of coal production including production, reserves, coal types, impurities, mining methods, and coal cleaning processes were shown in Table 3. In some coal mines where the coal quality is good, only crushing and screening are used while in others, coal cleaning like gravity separation (e.g., dense medium separation and spirals) and flotation are required. In terms of usage, about 80% of coal produced worldwide by the top 5 coal producing countries are processed by gravity separation techniques like jig, dense medium cyclone, and spirals (Fig. 3; [67]).

**Table 3:** Information of the largest mine in top 5 countries of coal production.

Rank	Country	Mine	Production [MTPA]	Reserves [BT]	Coal type	Impurities	Mining methods	Coal cleaning methods	References
1	China	Haerwusu coal mine	20.00	1.70	Lignite	anatase, bauxite boehmite, calcite clausthalite, kaolinite, lithium, pyrite, and zircon	open pit	dense medium separation	[[71], [72], [73], [74], [75]]
2	USA	North Antelope Rochelle mine	59.97	1.90	Subbituminous	anhydrite, bentonite, gypsum, and uranium	open pit	–	[76]
3	India	Jharia coalfield	0.34	19.40	Bituminous	amphibole, apatite, diopside, K-feldspar olivine, and phlogopite	open pit and underground	flotation	[[77], [78], [79]]
4	Australia	Peak Downs coal mine	11.80	0.72	Bituminous	copper, pyrite, and zinc	open pit	dense medium separation, spirals, and flotation	[80,81]
5	Indonesia	Sangatta mine	51.72	0.92	Lignite	illite, illite-montmorilonite, kaolinite, melanterite, quartz, and siderite	open pit	dense medium separation	[82,83]

This review seeks to answer the following research questions:

i. What is the current state of gravity separation technologies for coal cleaning?
ii. How has gravity separation technologies for coal cleaning progressed in the last 10 years?
iii. How can the advances in gravity separation for coal cleaning be utilized and applied to address challenges in other fields like environmental pollution and mitigation, waste recycling and reprocessing, circular economy, and mineral processing?

The literature was systematically reviewed to answer this question using the Preferred Reporting Items for Systematic Reviews and Meta-Analyses (PRISMA) guidelines [68] and the guidelines recommended by Andrews (2005) to identify peer-reviewed journal publications that reported about “coal cleaning” and its synonyms, including “coal concentration”, “coal preparation”, “coal processing”, “coal beneficiation”, “coal separation” and “coal washing” together with technical keywords including “wet”, “dry”, “coarse”, “fine size”, “shape”, “density”, “settling velocity”, “gravity”, “centrifugal”, “jig”, “dense medium”, “dense media”, “cyclone”, “spiral”, “table”, and “fluidized bed” [69]. Web of Science and Scopus were selected as databases for this systematic review, and the publication dates were limited to between 2010 and 2020 (i.e., 5 years before and after the ratification of UN-SDGs in 2015). Also, only peer-reviewed articles written in English were included [70]. Based on these initial criteria, a total of 1864 articles were screened after removing duplicates (Fig. S1). After a thorough evaluation (see Supplementary materials for details), the number of articles was reduced to 189, which were reviewed and summarized to evaluate recent research trends and progress about conventional gravity separation (Section 3), dry-type separation (Section 4) and enhanced gravity separation (Section 5). Finally, gravity separation challenges and future applications in environmental pollution and mitigation, waste recycling and reprocessing, circular economy, and mineral processing are explored (Section 6).

## 2 Overview of conventional coal cleaning technologies

Before going into the details of gravity separation for coal cleaning, an overview of conventional coal cleaning currently used in the coal industry is needed. In coal cleaning, the main goal of separation is to increase combustible organic materials and reduce inorganic gangue minerals, which has two benefits: (i) it increases the coal heating value (i.e., better quality), and (ii) it limits environmental emissions during combustion.

**Fig. 4:**
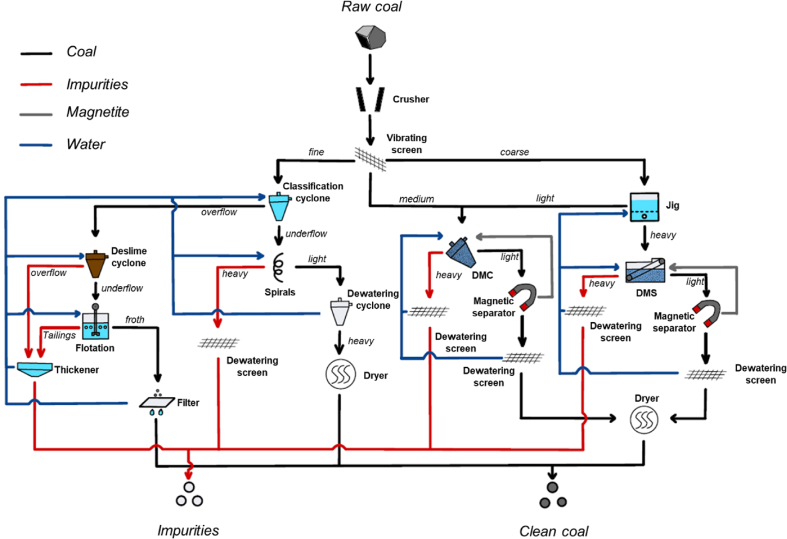
A typical schematic flowchart of coal cleaning.

**Fig. 5:**
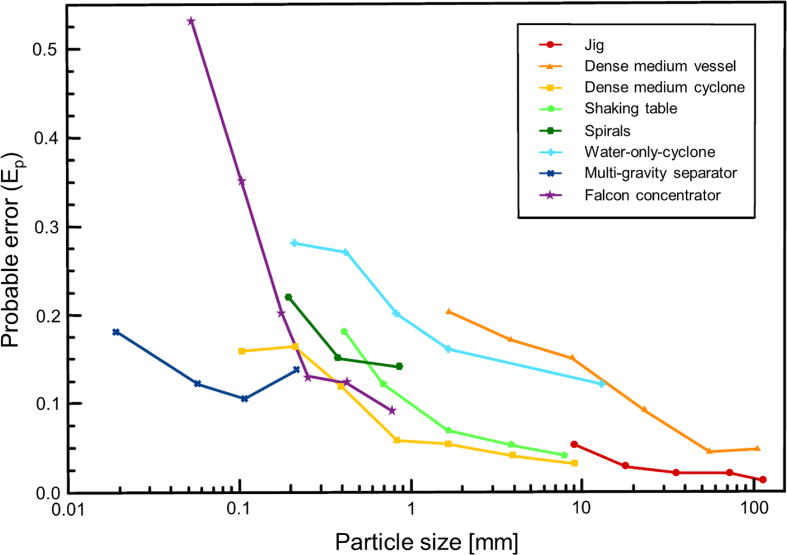
Particle size dependence of probable error (E_p_) of each coal cleaning method [67].

Fig. 4 shows a typical schematic flowchart of coal cleaning, which starts with size reduction of ROM coals. Coal comminution is carefully controlled to limit the generation of fine materials because the separation of fine particles is more difficult (Fig. 5). The probable error (E_p_) or Ecart probable moyen (E_pm_) is a parameter used to evaluate separation efficiency; that is, lower E_p_ values show higher separation efficiency [67]. Jaw crushers, hammer mills, and sizers (including single roll and double roll types) are choices available in coal cleaning plants [15]. Apart from these, rotary coal breaker—a machine that combines size reduction, screening, and separation—has been developed and specifically designed for selective coal comminution. Undersized coal can pass through a cylinder tumbling screen while unbroken gangue particles remain in the drum and are finally discharged at the downstream end [15]. After size reduction, screening—the easiest and most efficient method for sizing of coarse particles—is typically applied using grizzlies, inclined vibratory screens, high frequency screens, and banana screens. For fine particles, hydraulic classification using hydrocyclones is generally preferred. After crushing and sizing, more thorough separation of coal and gangue minerals is carried out using one or more of the methods listed in Fig. 5 depending on the particle size distribution of materials.

In most coal cleaning plants, separation frequently starts with gravity separation—a technique that separates particles based on differences in density—because of its operational simplicity, low cost, and high efficiency. For example, jig and static dense medium separation are commonly applied for coarse particles while dynamic dense medium separation, shaking table, and spirals are used for fine particles (more details will be described in Sections 3, 4, and 5).

Other separation techniques are also alternatively utilized depending on the situation. Flotation [41,84] is a surface-based separation that separates hydrophobic coal from hydrophilic gangue minerals by surface wettability difference. Since coal is hydrophobic, it tends to attach on air bubbles, which then carry it to the water surface for collection. Meanwhile, hydrophilic impurities especially silicate group minerals like quartz and feldspar remain in the water and are disposed of as tailings. For fine particles, column flotation [85] is more efficient compared to mechanical flotation because smaller bubbles could improve the probability of bubble-particle collision and the washing water could reduce the entrainment of fine gangue minerals. In some cases, carrier flotation might be used. Oil agglomeration [85,86]—another kind of surface-based separation technique—is appropriate for ultra-fine coal cleaning when flotation is ineffective. By adding oil, coal could be selectively agglomerated and separated from gangue minerals by simple screening [87].

Magnetic and electrical separation is applied in coal cleaning when the gangue is composed of magnetic and/or conductive minerals [88]. Iron oxides/oxyhydroxides, for example, can be separated from non-magnetic coal because they react to a magnetic field; that is magnetite is ferrimagnetic, goethite and hematite are antiferromagnetic, and lepidocrocite is paramagnetic [[89], [90], [91], [92]]. Magnetic separation may also be applied to remove oxidized pyrite that was treated with microwave [88,93]. Finally, dewatering using thickeners and/or by filtration is utilized to remove excess water and prevent handling, freezing and logistical problems [15].

## 3 Conventional gravity separation for coal cleaning

### 3.1 Pulsation-type technologies

Jigging is one of the oldest methods that is still widely used for coal cleaning. In a jig, the separation of minerals of different specific gravity is accomplished in a bed that is rendered fluid by a pulsating water current to induce stratification. The aim is to “fluidize” the bed of material being treated, controlling dilation so that the lighter, smaller particles penetrate the interstices of the bed while the larger, high specific gravity particles fall under a condition similar to hindered settling. It is usually suitable for coarse particles (Fig. 5).

**Fig. 6:**
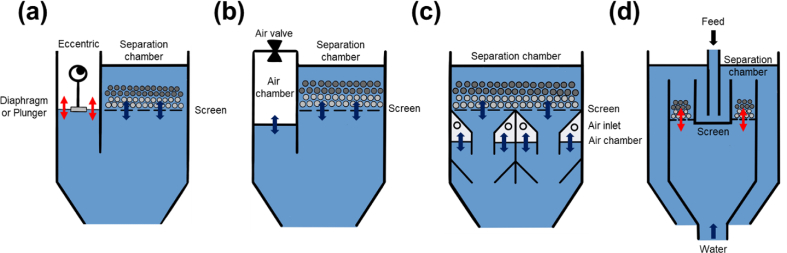
Schematic illustrations of (a) Denver or Harz jig, (b) Baum jig, (c) Batac jig, and (d) In-line pressure jig. Note: Black arrows mean the material flows, red arrows mean mechanical motions, and blue arrows mean the water flows. (For interpretation of the references to colour in this figure legend, the reader is referred to the Web version of this article.)

From a recent literature review, jigs that are currently used in laboratories can be divided into 2 types based on the configuration of jig sieve: (i) fixed screen, and (ii) moving screen. For fixed screen jigs, Denver or Harz (Fig. 6a), Baum (Fig. 6b), and Batac jigs (Fig. 6c) are used [[94], [95], [96], [97], [98]]. The Denver jig is applied more for the separation of ores in mineral processing and only found limited application in coal cleaning. Baum and Batac jigs—the air pulsation types—are more commonly used for coal cleaning because air pulsation provides better water pulsation control than mechanical-type diaphragm used in Denver jig (Fig. 6a) [99,100]. Furthermore, Baum and Batac jigs are capable of over the screen and through the screen methods for the extraction of heavy gangue minerals while the Denver jig is limited only to through the screen recovery. In a Baum jig, the air chamber is located next to the separation chamber (Fig. 6b), however, in Batac jigs (originally called TACUB (Takakuwa air chamber under bed) jig), the air chambers (single or multiple but, usually 2 cells) are positioned under the separation chamber (Fig. 6c). For moving screen jigs, in-line pressure jig (IPJ) with mechanical pulsation mechanisms and through the screen recovery method is the most common example [101] (Fig. 6d).

Recent studies about jig separation are looking into the effects of various parameters including inherently controllable (manipulated variables) and uncontrollable factors (disturbance variables) [94,97,102,103]. Moreover, studies on how to modify and apply jig separation for fine coal particles are increasing [98,103,104]. There are many recent studies about improvement of jigging performance by mathematic modeling, simulation of particle motion, and process monitoring methods like statistical optimization [98], computational fluid dynamic and discrete element method (CFD-DEM) model [101], dynamic model using Matlab/Simulink software [105], 3D response surface approach [95,96], on-line video analysis [106], and radiometric density meter [[107], [108], [109], [110], [111]] (Table S1).

### 3.2 Flowing film-type technologies

**Fig. 7:**
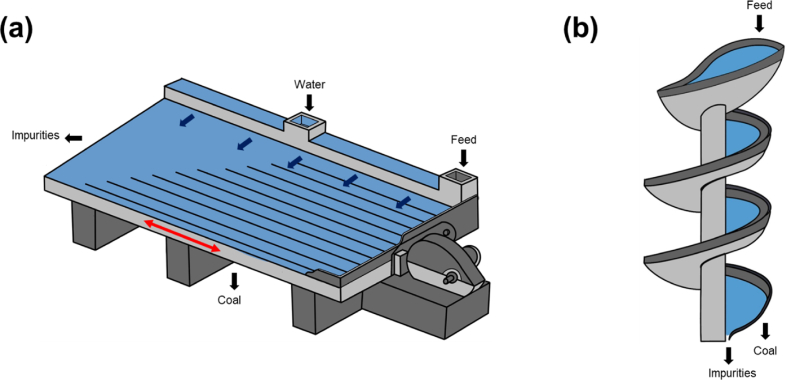
Schematic illustrations of (a) shaking table and (b) spiral concentrator. Note: Black arrows mean the material flows, red arrows mean mechanical motions, and blue arrows mean the water flows. (For interpretation of the references to colour in this figure legend, the reader is referred to the Web version of this article.)

Shaking table (Fig. 7a)), —a flowing film-type gravity separator—is one of the most versatile devices for mineral processing. Shaking tables are characterized by low power consumption and low costs of operation, installation, and maintenance. However, low capacity and large installation area are its disadvantages. The E_p_ of shaking tables is lower when compared to spirals and water-only-cyclone (WOC) that are usually used for similar size fraction of particles, indicating better separation efficiency (Fig. 5). It is preferable for coal particles between 0.5 and 5 mm in size. In some cases, however, it may be applicable down to 0.075 mm and up to 15 mm size fractions.

A shaking table contains a slightly inclined deck. Feed is introduced at the feed box and is distributed across the table by a combination of table motion and water flow. The multilayer is introduced onto the table and vertical stratification due to shaking action takes place behind the riffles. Coal with lower SG is carried by water flow over the riffle and is recovered as the light product while heavier gangue minerals are trapped behind the riffles and moved to the heavy product launder due to the shaking motion of the table.

In recent years there are only a few papers published about coal cleaning using shaking tables [102,112,113]. Shahzad and Ali (2018), for example, evaluated the effects of table slope and water flow rate on coal cleaning and found that increasing the table slope and water flow rate could increase yield but increases the ash content of coal in the light products [112].

Spirals, also known as spiral concentrators (Fig. 7b), are flowing film-type separation tools that stratifies particles by gravity. Humphrey's coal spirals are commonly used to process coal in the size range of approximately 0.15–1.0 mm. It consists of a corkscrew-shaped device that selectively segregates coal from waste rock when particles move in the flowing film along the helical trough [114]. The mixture of water and ground particles is gravity fed from the top then flow spirally downwards. The particles stratify due to the combined effects of centrifugal force—the differential settling rates of the particles—and interstitial trickling through the flowing particle bed. Coal is recovered in the “edges” as the light product while the heavier impurities are recovered towards the center of the spirals [102]. The efficiency of spirals and the quality of the resulting products can be improved by removing fines through screening and/or classification (e.g., classifying cyclones, Pansep screen, and especially stack sizer) [99,[115], [116], [117]].

Spirals are often employed in combination with other water-based separators (e.g., flotation) to improve the performance [116]. Automation and control system were applied on coal spirals with real-time sensors and adjustable splitter to change the cut point of SG and increase coal yield in real time [114]. Another application of spirals is in the pre-concentration to remove impurities such as muscovite and illite from stone coal to improve vanadium recovery [118].

### 3.3 Sink-and-float separation-type technologies

Dense medium separation (DMS)—also known as heavy medium separation (HMS), heavy liquid separation (HLS), or sink-and-float separation—separates two materials with different densities using a fluid medium with a density intermediate between the two materials. This causes the lighter particles (e.g., coal) to float while the heavier materials (e.g., shale or high-ash coal) sink. The medium commonly used is a suspension of fine magnetite in water which could be recirculated after washing, magnetic separation, and demagnetization. In this review, only articles with DMS are included while those about sink-and-float analysis and/or washability curves without DMS are excluded.

**Fig. 8:**
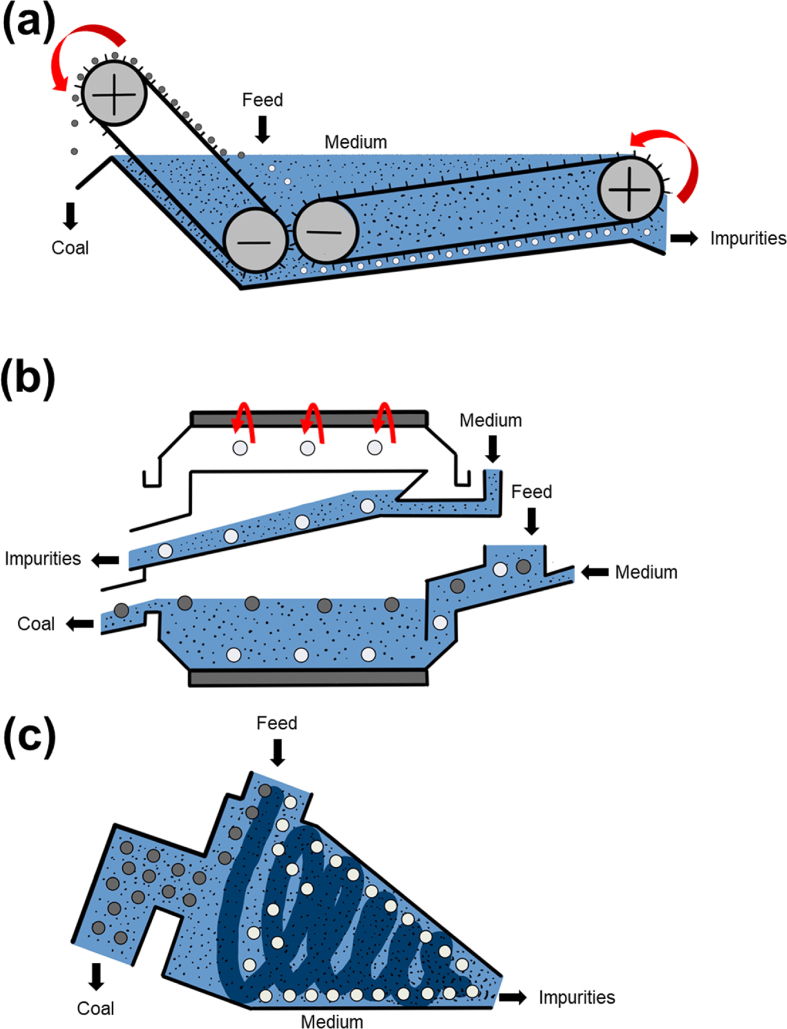
Schematic illustrations of (a) dense medium bath, (b) dense medium drum, and (c) dense medium cyclone. Note: Black arrows mean the material flows and red arrows mean mechanical motions. (For interpretation of the references to colour in this figure legend, the reader is referred to the Web version of this article.)

DMS is classified into two general categories based on the separation vessel: (i) static or gravitational, and (ii) dynamic or centrifugal. Static-type DMS separates particles by gravity only while the medium is static and is suitable for particles greater than 12.5 mm. Two types of vessels—bath and drum—are used for static-type DMS. The dense medium bath (DMB) (Fig. 8a) is a large open tank with the circulation of finely pulverized magnetite suspension while the dense medium drum (DMD) (Fig. 8b) is a rotating longitudinal cylindrical vessel where waste rocks sink and are collected by lifters to be discharged into the launder [119].

Two types of cyclones are used in gravity separation of coal cleaning: dense medium cyclone (DMC) (Fig. 8c) and water-only-cyclone (WOC). DMC is costlier than WOC but can achieve sharper separations than WOC, making it suitable for cleaning coal containing gangue minerals with similar density as coal. Fine particle (0.5–8.0 mm) separation can be achieved in dynamic separators because centrifugal force is more effective than gravitational force. More recently, DMC has become widely used in coal cleaning. The principle of operation is similar to that of hydrocyclone (classifying cyclone). The feed is suspended in the medium and introduced tangentially to the cyclone. The heavy impurities are centrifuged to the cyclone wall and exits at the apex while light coal “float” to the vertical flow around the axis and exit via the vortex finder [120,121].

Only a few studies for DMB and DMD were carried out while many studies on DMC have been done. Recent studies on DMB and DMC have been focusing on yield optimization and determination of optimum separation densities for coarse and fine particles using Solver and Mathcad [122,123]. For DMD, Meyer and Craig (2015) proposed the dynamic model for dynamic control of the Wemco drum to estimate the grade of products using coal washability and drum partitioning behavior [119]. A follow-up work of these authors in 2016, applied a non-linear model predictive control (NMPC) to increase yield and minimize ash content of coal products by simulation [124].

For DMC, the studies are summarized in Table S2. For works about cyclone vessel, the development of a three-product DMC—2 stages DMC consists of cylindrical DMC connected with conventional conical DMC [125,126]—and Tri-Flo separator—2 stages cylindrical DMC [127,128]—are noteworthy. For the separation media, silica-based materials [129] and medium composites [130] have been evaluated as alternatives to magnetite in DMC. Modeling and simulation are the most popular topics for DMC. Computational fluid dynamic (CFD) is the most widely used [131] and is usually combined with other techniques like discrete element method (CFD-DEM) model [[132], [133], [134], [135]]. Also, the development and application of dynamic model [136], dynamic and steady-state model [137], Rosin-Rammler model [138], and soft sensor model [120] have been reported. System controls (i.e., smart sensor [130], model-data-based switching adaptive control [139], pump-storage system [140], medium density control [141], dual-loop control system [142], and intelligent control [143]) were also applied to measure and optimize separation. In addition, DMC has been evaluated for the reprocessing of coal tailings [144], including the effects of near gravity materials (NGM)—the percentage of material occurring within the range of ±0.1 cut density (CD)—on coal cleaning [145].

### 3.4 Other technologies

For very fine particles with sizes close to the particle size of dense medium, a water-only-cyclone (WOC) is used instead of a washing screen. Similar to DMC and hydrocyclone (classifying cyclone), centrifugal force accelerates the settling rate of particles thereby separating particles based on size, shape, and SG [146]. Particles with faster settling velocity (i.e., gangue minerals) move to the wall of the cyclone, where velocity is the lowest, and then migrate to the apex opening. Due to drag force, the slower-settling particles (i.e., coal) move towards the zone of low pressure along the axis and are carried upward as overflow through the vortex-finder.

Compared to DMC, WOC is cheaper since the heavy medium is not needed, and the preparation and recovery process associated with heavy medium are not required. An autogenous (self-generated) dense medium could be derived from heavy gangue mineral contained in coal which enhance gravity separation using water. Because of its simplicity, small space requirements, lack of moving parts, low operating costs, low maintenance requirements without dense medium, the WOC is widely used in coal beneficiation [147].

WOCs are similar to a hydrocyclone in design and operation, but they are configured to separate materials on the basis of density instead of size. In general, classification (sizing) occurs in the free settling regime while density separation takes place in the hindered settling regime. The modified geometry of WOCs (wild angle conical bottom) compared to conventional hydrocyclones is responsible for creating an autogenous bed of particles along the conical section of the cyclone, where hindered settling dominates [148].

Because WOCs are regarded as affordable separation devices, many recent studies tried to improve their separation efficiency for fine coal cleaning. The principle and the model of WOC are similar to the classifying cyclone; that is, size classification occurs during gravity separation especially when the SG difference between coal and gangue mineral is small and their size fraction difference is wide [148]. Hembrom and Suresh (2018) investigated the effects of outlet dimensions of WOC on vortex finder length (VFL), vortex finder diameter (VFD), and spigot diameter (SPD), and reported that the order of significance of the variables was found to be VFL > SPD > VFD for % Yield and VFL > VFD > SPD for % ash [149].

Many modifications to WOC for coal cleaning have been studied. For example, Hacifazlioglu (2012) developed a modified WOC (MWOC). It was found that MWOC produced clean coal of equal quality when compared with spirals and flotation but with a lower %yield [146]. Other examples of modification of WOC include dual cone WOC [150], three-product cyclone or two-stage cyclone [147,151], and WOC with three-stage cone [152]. In addition, data modeling using CFD has been applied to WOC for improved yield and efficiency [153].

Fluidized bed separators (FBS)—also called teeter bed separators (TBS) and hindered-bed separators—are initially used for size separation and can be operated to provide efficient density-based separation for fine-sized fraction by taking advantage of hindered settling and autogenous dense medium that is naturally generated from the fine and heavy impurities, as described earlier in WOC. Feed slurry is fed into the vessel and the separation occurred by the settlement of particles in the upward current of water flow. Fluidization is a phenomenon where solid particles are suspended in an upward flowing fluid and behave as a pseudo fluid. The velocity of rising water should be controlled to match with the finest fraction of heavy particles to form the fluidized bed. Light coal particles cannot penetrate the bed and are recovered as the overflow while heavy gangue minerals will be recovered as underflow products and/or became the fluidized bed depending on their settling velocities [154]. The principle of FBS was utilized and modified into various techniques as described below.

Floatex density separator (FDS)—also called counter-current, and autogenous teetered bed separator—is an advanced hindered settling classifier which utilize particle settling rate to segregate different particles according to size, shape, and density. Feed slurry is introduced into the FDS through a centralized feed and fluidized (teeter) water is introduced from the base. Feed is expanded into teeter or fluidized bed due to the rising current of water. After that, the separation will occur. Solid particles in the feed slurry settle down under the gravitational field against the rising current of water. The heavy gangue particles settle down to form a bed while lighter coals are carried to the overflow stream [155,156]. In coal cleaning, FDS is used as a pre-concentrator and/or a classifier prior to other separation processes. Teeter water flowrate, bed pressure, set-point, feed rate, and pulp density are the important parameters affecting separation [155,156]. It was also found that higher bed pressure and higher teeter water flowrate could enhance the yield of clean coal albeit with poorer grade [156].

CrossFlow separator is another important development in hydraulic separation that is more efficient and has high capacity in processing fine coals. The main reason is due to more quiescent flow regime in the teeter chamber. Feed is gently introduced across the top of the chamber, leaving the chamber contents largely undisturbed. The upward velocity in the separator is more constant since the feed does not directly enter to the teeter bed resulting in high separation efficiency. Only few studies about CrossFlow separator have been done. For example, Hansen-Carlson and Das (2019) studied about the settling characteristics and separation features for the cleaning of fine coal using a laboratory scale CrossFlow separator and recommended that a quiescent flow must be maintained for the better performance [157].

Allflux separator is also a kind of separator that applies the principle of liquid-solid fluidization. The allflux separator consists of a coarse and fine separation chamber. The feed is introduced into the coarse chamber, then the overflow goes to fine chamber and the overflow of fine chamber is collected as the final product, resulting in three fractions: (i) concentrate (clean coal), (ii) middling (fine underflow), and (iii) tailings (coarse underflow) [158]. This separator is another separator about which only few studies were published, i.e., Tripathy et al. (2016) that used statistical model to optimize the cleaning of high ash semi-coking coal [158].

Reflux classifier (RC)—one of the most recent developed separators that is widely utilized in coal cleaning—is a combination of liquid fluidized bed, autogenous dense medium and lamella settler. RC consists of a set of parallel inclined channels located above a vertical section with fluidizing water added through the base. The FBS acts as the primary density-based separator whereas the lamella section acts the secondary separation zone in the RC. In the fluidization section, rising fluidization water keeps incoming coal particles in the fluidized condition and these particles settle against the fluidization water similar to the separators explained earlier. The fluidization section provides a uniform flow of particles to each inclined plate in lamella section. Laminar flow could be promoted by a closely spaced channel due to laminar-shear mechanism that make the segregation of fine gangue particles become packed in the fluidization chamber while light coals are transported upwards to the overflow. This phenomenon develops a reflux action because of fluidized particles segregating onto the inclined plates and returning to the fluidized zone below. This self-recycling effect eliminates the misplacement of materials, thus enhancing the product quality. RC has several advantages over conventional FBS, spirals, and DMC which includes higher separation efficiency, robust operation, less floor space requirement, consistency in product grade and high throughput. Inclined channels have a higher throughput capacity compared to a conventional FBS due to their increased effective settling area, so-called Boycott effect [[159], [160], [161], [162]].

There are many recent studies about RC. Kopparthi et al. (2019) reported that the ash content and yield could be increased by increasing the water rising velocity and bed setpoint [162]. It was also confirmed that the separation of RC was partially dependent on particle size; that is, only for coarse size fraction with large channel is size independent while for fine particles, particle size has a significant effect on separation efficiency. To suppress the effects of particle size, more closely spaced inclined channel is preferred for fine particles because this configuration promotes laminar flow, resulting in better separation efficiency (lower E_p_) [159,163]. Two-stage gravity-deslime RC was also proposed [160,164]. In addition, it was also available to be applied with other separation techniques like spirals, DMC, and flotation [160,165].

Inclined tapered diameter separation bed (ITDSB), a novel separator that was initially designed for metal recovery from waste printed circuit boards (WPCBs), was applied for coarse coal slime separation [166]. ITDSB uses water as a medium and was devised based on the theory of inclined flow separation as explained earlier in RC. The CFD-DEM model has been proposed for particle simulation and distribution of coarse coal slime in ITDSB. Based on the model, a quadratic function of combustible recovery and obliquity, water flow, and feed grade was suggested [166].

## 4 Dry-type gravity separation technologies

Coal cleaning is mostly carried out by wet processes due to their high separation efficiency. However, wet processes consume large amount of water that are difficult for dry areas with limited water supply as well as the cold areas that are the temperature below the freezing point of water. Considering these problems as well as systems control, the application of dry-type coal beneficiation methods, such as fluidized bed, air jig, air table, and negative pressures pneumatic separator (NPPS) [167] have been developed [168]. It was also applied to separate coal from near gravity materials (NGM) [169] as well as heavy metal like mercury (Hg) [170,171] and arsenic (As) [168]. Recently, most of the published articles related to gravity separation for coal cleaning are about dry-type processes and most of the researchers are from China since more than two-third of coals in China are distributed in arid and water scarce areas [172,173].

### 4.1 Fluidized separation with dense medium technology

**Fig. 9:**
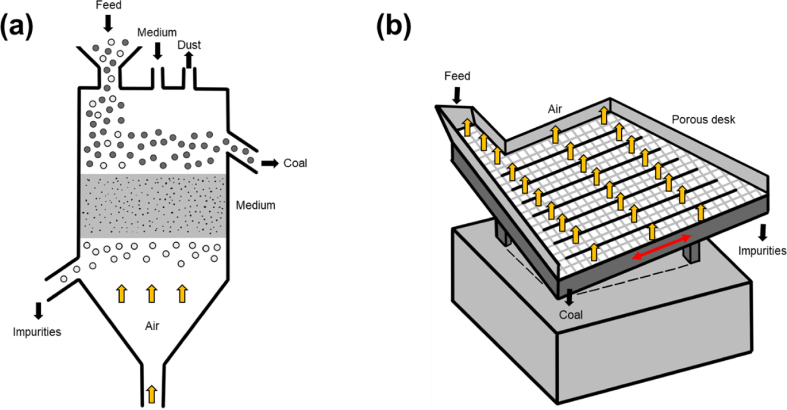
Schematic illustrations of (a) air fluidized bed separator and (b) air table. Note: Black arrows mean the material flows, red arrows mean mechanical motions, and yellow arrows mean the air flows. (For interpretation of the references to colour in this figure legend, the reader is referred to the Web version of this article.)

Air dense medium fluidized bed (ADMFB) separation is the most widely used technology for dry-type coal cleaning with the efficiency comparable to those of wet-type methods. In an ADMFB, a solid medium transforms from a packed bed to a fluid-like state by an upward air stream. The pseudo-fluid behavior of gas-solid fluidized bed is utilized to separate the immersed feed coal, and clean coal with density less than the density of the fluidized bed float on the top of the bed, whereas the heavier ones (gangues) settle towards the bottom and are then rejected as tailing product (Fig. 9a).

**Fig. 10:**
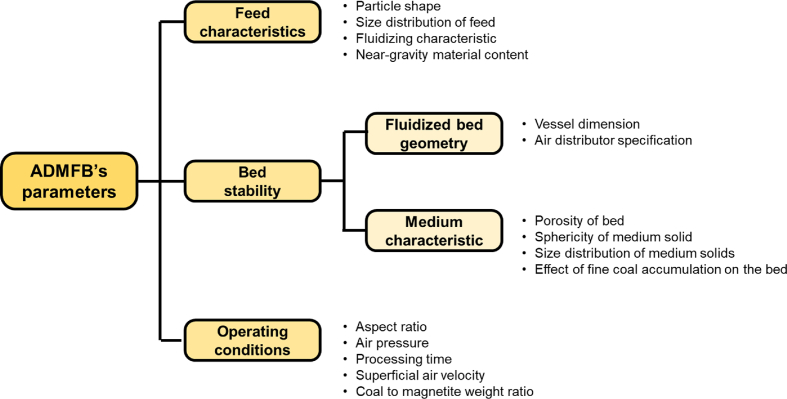
Schematic diagram of important parameters that affect the separation efficiency of ADMFB [242].

Generally, magnetite particles are used as the medium material because they can be recycled easily using their magnetic property, thus avoiding medium particle loss during coal separation. Lower upward air flow rates and smaller dust-collecting equipment with minimum possible moving parts and the possibility of using waste heat (low-quality heat) for simultaneous coal drying are some of the advantages of the ADMFB separators over the other dry methods [[174], [175], [176]]. Most of studies related to gravity separation in coal cleaning are from China and focused on ADMFB. Fig. 10 and Table 4 show the details of important parameters from these recent works.

**Table 4:** Important parameters of ADMFB in coal cleaning.

Parameter	Description	Reference
Density of impurities	The increase of density difference between coal and gangue minerals could increase the separation performance.	[176]
Particle size	The separation of large particles is better than that of fine particle and size classification is usually required to prevent the segregation by size.	[57,[216], [217], [218], [219], [220]]
Particle shape	Shape of feed could also affect the separation efficiency. Sphere-like shape showed better results followed by rod-like shape while disk-like shape showed the worst results. Since the settling velocities of irregular shapes are higher than that of spherical shape, high-ash particles with non-spherical shape settle down difficultly and were collected with coal products.	[173]
Feed rate	Decreasing feed rate could increase the separation efficiency	[221]
Superficial gas velocity	Decreasing air velocity leads to the increase of density stratification, however, appropriate gas velocity could be controlled.	[57,173,176,180,184,193,222,223]
Fluidized bed height	Reduction of bed height slightly increased density stratification	[176,184]
Coal to magnetite weight ratio	Higher amount of magnetite resulted in higher separation efficiency.	[218,224]
Size distribution of medium solid	Suitable size fraction of magnetite (e.g., +75–106 μm) should be controlled.	[225,226]
Medium density	Higher bulk density of medium resulted in higher separation efficiency.	[188]
Time	Increasing time results in the increase of segregation efficiency	[227,228]
Bed height	Increasing the bed height could lower the fluidization performance and cause lower separation efficiency	[57,180,196,222,223]
Bubble size	Smaller bubble size results in lower ash content in the product.	[229]

Many of these studies evaluated the application of unconventional media like paigeite powder [177] and binary medium—the mixture of magnetite with other minerals such as pyrrhotite or quartz [168,[178], [179]]—instead of conventional pure magnetite.

Similar to wet-type DMS like DMC, modeling and simulation are popular themes for ADMFB. Most of the models were based on CFD and a Eulerian-Eulerian multiphase flow model or two fluid method (TFM) [57,[180], [181], [182]]. TFM-DEM hybrid model, the combination of TFM and discrete element method (DEM), was the most popular one. The gas phase and medium solid phase are modeled by TFM while fine coal particles are modeled by DEM [[183], [184], [185]]. Other models like magnetite particle surface attrition model [186], the Syamial-O’Brien drag model [187] and the disperses k-ϵ turbulence model [187] were also used.

These studies were carried out using various monitoring systems or equipment like densimeter [188], flowmeter or rotameter [173,[188], [189], [190], [191], [192], [193], [194], [195], [196], [197]], manometer [[188], [189], [190],^193^,^194^,^196^,^198^], pressure meter [188,190,191,197,199] and high speed camera [173,193,195,196,199]. In addition, systems control (i.e., density control [200,201] and flow control [195] were applied to measure and optimize separation.

More recently, vibrated fluidized bed (VFB) and pulsation fluidized bed (PFB), which can strengthen the movement of particles, increase gas-solid contact rate, improve fluidization quality, and achieve fluidization of fine cohesive particles are applied for coal cleaning [202,203]. Vibrated dense medium fluidized bed (VDMFB) [[203], [204], [205], [206]], gas-vibro fluidized bed (GVFB) [197,207,208], and pulsating air dense medium fluidized bed (PADMFB) [202,209,210] are the examples of these technologies. In these types of ADMFB, vibration energy weakened the influence of bubble that occurred in conventional ADMFB since the fluidization stability could flexibly adjust by the pulsating airflow [204,205]. However, information about these is still limited. Other techniques like tapered fluidized bed [211], a novel ADMFB with wear-resistant, anti-blocking air distributor plate, and novel discharge device [212], secondary air-distribution fluidized bed separator (SADFBS) [213], pronation-grille baffle dense phase medium fluidize bed [214], and porous sponge fluidized bed (PSFB) [215] were also used.

### 4.2 Fluidized separation technologies without dense media

Similar to ADMFB separator, the fluidized separation could also be achieved without dense medium by the generation of an autogenous medium occurred from the fine gangue minerals under the fluidization by air like explained earlier in wet fluidized bed separation.

Vibrated fluidized bed separator was developed for density segregation in the field of gas–solid fluidization for fine coal dry beneficiation [230,231]. Utilization of a vibrated fluidized bed could intensify the density segregation within the bed of fine coal, leading to an effective separation performance. Particles in the bubbling-region considerably lower solid concentration than the surrounding bulk phase. The sink-float separation by pseudo-fluid of autogenous medium let the higher density impurities sink while lighter coal float. The experimental results of many researchers showed that superficial air velocity was the major parameter affecting the separation [[231], [232], [233], [234], [235]], followed by vibration (i.e., amplitude [230,231,233,234] and frequency [230,231,[233], [234], [235]]) and other parameters (i.e., feed rate [236] and separation time [230]) while bed height did not significantly affect the separation [231,234] (Table S3). Recently, ultrasonic vibration gas-solid fluidized bed was developed to separate coal and high-density impurities like pyrite, quartz, and kaolinite using ultrasonic vibration force field [237].

Air jig or pneumatic jig, a deep-bed separator that deploys the difference in hindered settling velocities of particles with different density, was developed using the same principles as wet jig. Air is supplied into the jig in two forms—a continuous flow and a superimposed pulsated airflow—that provides the impetus for stratification and consolidation trickling.

Stump air flow jig and Allair jig are two of the most commonly used air jigs in coal cleaning. Boylu et al. (2015) studied the operational parameters of Allair jig to optimize its separation efficiency, and the results showed that separation efficiency was enhanced by increasing pulsation frequency, reducing air velocity, and decreasing the discharge stargate rate [238].

Similar to air jigs, pulsating airflow classifier (PAC), a kind of vertical air classifier with sine-like shape air pulsation [239], and active pulsing air separation system (APSS), a system consists of a separation column and other assisted equipment to generate air pulsation with the harmonic waveform [240], were developed and utilized in coal cleaning using the separation/classification induced by air pulsation. Effects of the characteristics of air pulsation (i.e., amplitude and frequency) and feed rate were also investigated, and similar results were found. In addition, air-fluidized Reflux Classifier (RC) with the principle similar to conventional RC was also applied [241].

### 4.3 Compound dry-type separation technologies

Air or pneumatic table (Fig. 9b) is initially the most common dry gravity separator. This dry table uses the similar motion to wet shaking table. Air tables employ the stratification of materials by a combination of fluidizing air flow and other principles. The perforated deck is inclined in the longitudinal (end tilt) as well as transverse direction (side tilt). The fluidization of the material on the deck surface is achieved by blowing air from the bottom of the deck. The air effectively stratifies the bed material vertically, with heavy particles at the bottom and light particles on top. The vibration forces work on the stratified bed to achieve segregation on the deck plane. Heavier particles drop to the bottom of the table, where further movement is hindered by riffles, and travel in the direction of the deck's vibration. The lighter particles (combustibles) are lifted by the fluidizing air and assisted by gravity travel down the slope towards the discharge end. At the end of the deck in the longitudinal direction, the products are separated by the adjustable splitters [[243], [244], [245]].

Currently, Fuhe Ganfa Xuan mei (FGX) separator is the most well-known separator with high efficiency for coal separation in China [246,247]. The FGX separator consists of a perforated inclined deck with several air chambers (usually three), a vibrating device, a drive and a mechanism which allows the change of table inclination angle. The separating deck with riffles, is suspended inclined both in longitudinal and transverse directions similar to the conventional air table. The method also utilizes integrated effects of vertical and horizontal segregation by air fluidization and table vibration, respectively. Airflow from a centrifugal blower goes through the perforated deck (usually different air flow rates could be adjusted for each air chamber) and forms a rising air current then fluidizes feed material on the deck. The fines (−1 mm) present in the feed could generate an autogenous separating medium due to this air flow. This pseudo fluid could enhance the separation by density using the sink-float mechanism that is better than that of conventional air table. The lighter particles go up through the surface of the suspension bed then turn inward towards the discharge point. While heavier particles gravitate through the bed and come in contact with the deck surface and move in the direction of the outlet of rock, is discharged by the baffle plate that directs them to the discharge chute of rock [175,[248], [249], [250]]. Particle size [[250], [251], [252], [253]], feed rate [246], air flow rate [249,256], table transverse inclination [246,256], table longitudinal inclination [246,256], table vibration frequency [246], riffle height [246], and partition plate height [248,[257], [258], [259]] are the parameters that could affect the separation efficiency of both conventional air table and FGX separator (Table S4). FGX separator could also be applied to remove heavy metals like mercury from hard coal [254] and used for low-rank lignite beneficiation with the combination of subsequent infrared (IR) drying [250]. Regression model using response surface methodology (RSM) was applied for FGX separator to statistically optimize the separation [255].

In addition, other compound dry-type separators, namely compound separator [246], table-type air separator [251], multi stag air table [260], and Kigam air table (KAT) [261], were also developed by applying the principles and designs of conventional air table as well as FGX separator.

## 5 Enhanced gravity separation technologies

Conventional gravity separation is ineffective in separating fine (<0.1 mm) coal and gangue minerals (Fig. 5) because the settling velocity difference under a normal gravitational field of 1G is small. Rather than gravity separation, surface-based separation techniques like flotation and oil agglomeration are commonly used. Another option to treat very fine coal is by applying an artificially enhanced gravitational field using centrifugal force to increase the setting velocity as well as settling velocity difference resulting the higher efficiency. Centrifugal separation, also known as enhanced gravity separation (EGS), are primarily developed for desulfurization by removing of fine pyrite (FeS_2_, SG 5.0) and has recently evolved to achieve efficient separation of fine gold particles on a commercial scale. EGS can be categorized into three groups: pulsation type, fluidized bed type, and flowing film type [262].

### 5.1 Pulsation-type technologies

**Fig. 11:**
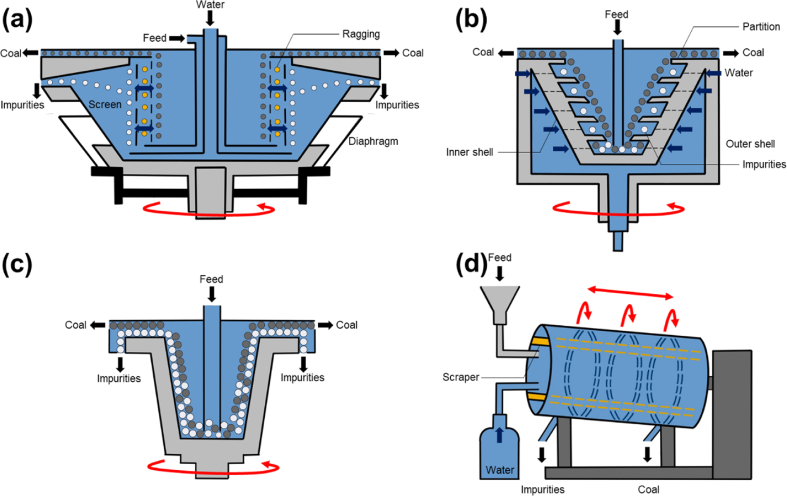
Schematic illustrations of (a) Kelsey jig, (b) Knelson concentrator, (c) Falcon concentrator, and (d) multi gravity separator. Note: Black arrows mean the material flows, red arrows mean mechanical motions, and blue arrows mean the water flows. (For interpretation of the references to colour in this figure legend, the reader is referred to the Web version of this article.)

A schematic diagram of a Kelsey centrifugal jig (KCJ) is shown in Fig. 11a. It works on the principles of a conventional jig employing a centrifugal force field to enable the treatment of ultrafine particle. The equipment is made up of a cylindrical screen that spins coaxially on a fixed central feed pipe by a rotating rotor that is capable of generating centrifugal fields up to 100G. Feed slurry enters through the pipe and flows outwards across the bed of ragging. Pressurized water is added to dilate the feed as well as ragging the bed to aid in stratification. Lighter coal flows through the surface of the ragging bed supported by a cylindrical screen that is mounted across the top of each hutch and overflows the top of the separator, while high-density minerals pass downwards through the ragging and screen which are discharged through actuated valves [262]. Due to better production capacity, simplicity, and less need for plant area, KCJ was chosen above other EGSs to use in the commercial scale [263].

The recent studies on KCJ are mainly related to optimization of parameters for better separation efficiency. Particle size, feed flow rate, ragging characteristics, spin frequency, stroke length, and pulsation rate are the important parameters of KCJ [262,263]. Shekhar et al. (2018) reported that pre-treatment using hydrocyclone for desliming prior to KCJ could reduce the ash content and increase the yield of clean coal compared to that of without pre-treatment [263].

However, another type of centrifugal jig like the Altair centrifugal jig which is simpler designed compared to Kelsey jig due to lower number of moving parts, does not have any studies related to it in recent years.

### 5.2 Fluidization-based technologies

The Knelson concentrator (KC), a compact centrifugal separator is a hindered settling device related to a hydrosizer, with an active fluidized bed to capture heavy minerals with centrifugal force equivalent to 200G [264]. It consists of a rotating truncated cone (bowl) which is stair-stepped by several ring-type partitions (Fig. 11b). Feed slurry is injected through the central feed pipe and is allowed to flow countercurrent between each partition until it overflows the top of the rotating bowl. Rinse water forced through perforations in the rotating bowl creates a fluidized bed of particles between each partition. Particles that have higher density than the fluidized bed are trapped in the retention zone between partitions while lighter particles are flushed out over these partitions. One of its disadvantages is the large amount of water (up to 2–3 times the feed flow) required to fluidize the particle bed [264].

Uslu et al. (2012) reported that particle size, blow speed, and fluidizing water pressure could affect the separation [264]. Özgen et al. (2019) proposed the equations as a function of gravitational force, solid concentration, fluidization water pressure, and sample collection time, to estimated ash content and recovery of clean coal product [265]. Similarly, Sabah and Koltka (2014) developed the regression model in the function blow speed, fluidization water pressure, sample collection time, and solid concentration, to estimate ash content of clean coal products [266]. When centrifugal force is low (low bowl speed), KC will work like a hydrocyclone which means the separation did not take places upon particle density but particle size. To optimize the separation of KC, high solid concentration, low fluidized water pressure, and high blow speed must be used. In this hindered settling condition, autogenous medium for fine and ultrafine clay particles will be generated and separation efficiency will be better [266].

### 5.3 Flowing film-based technologies

A Falcon concentrator is a spinning fluidized bed EGS which is basically a combination of a sluice and a continuous centrifuge having the highest centrifugal force of up to 300G. It enables the treatment of particles even down to 10 μm. Similar to Knelson concentrator, it consists of a fast-spinning smooth-surface truncated bowl (Fig. 11c). Fine coal is introduced from the top of the unit through a central vertical feed pipe. A thin flowing film of particles at its wall is formed and rapidly stratified based on their difference in density. Light particles like coal can move to the top layer and be discharged over the top of the cone, while heavy particles sliding along the inner surface of the cone are discharged through the cone wall [[267], [268], [269]].

Many researchers reported that particle size is an important parameter for Falcon concentrator. Screening and classification, for examples, single-desk full-scale Stack Sizer [270] hydrocyclone [269], as a pre-treatment could improve the separation efficiency of Falcon concentrator. The increase in particle size by flocculation using polyacrylamide (PAM) as a flocculant to enhance the difference of settling velocity was studied by Zhu et al. (2017) [271]. Their results showed that ultrafine coal with flocculant pre-treatment and artificial coal with the same size having similar sulfur content, ash content, combustible matter recovery, and desulfurization efficiency, indicating that the flocculation could enhance the separation performance of Falcon concentrator.

Not only particle size, another particle geometric aspect like particle shape could also affect Falcon concentration. Zhu et al. (2017) report that mismatched phenomena occurred as the existence of heavy particles in overflow, and light particles in the grooves could be resulted by increasing drag force due to the irregular shapes [272]. Other parameters like bowl speed (gravitational or centrifugal force), water pressure, solid concentration, and feed rate, also affect the separation efficiency of Falcon concentrator [269,271,273].

Oruç et al. (2010) derived the regression equation in a function of gravitational force, solid concentration, flow rate, and water pressure, by applying the least square method using Minitab 15 software [269]. Similarly, Boylu (2013) proposed response surface recreation method using Minitab 14 software to estimate separation efficiency, ash content in concentrate and ash content in tailing using the operating factors of centrifugal force and solid concentration [267]. The model was in-line with the experimental results and Boylu (2014) found that separation of Falcon concentrator could drastically change due to the solid concentration [268]. At low solid concentration (<10%), particle size-based separation occurred under free settling condition. At 10–40% solid density-based separation under hindered settling condition was done. And at higher solid concentration (50–55% depending on the particle size) an autogenous medium due to clayey minerals in feed could occur. However, this phenomenon also depended on the gravitational or centrifugal force.

A multi-gravity separator (MGS) (Fig. 11d) combines the normal gravitational pull of oscillating motion (4–6 cycles per second) of the drum similar to the shaking table and centrifugal force (90–150 rpm, about 5–15G) from the rotating motion of drum to provide an enhanced gravity separation of the fine particles. The feed, which is usually the underflow product of hydrocyclone, is introduced into the tilt-equipped drum. While the drum is rotating, the centrifugal force from drum rotation affects gravity separation so that coarse particles or high density, high ash content, are spiraled outside and settled on drum surface then slowly swiped up by scrapers to be discharged at the outer end. Meanwhile, the fine or light density particles less affected by centrifugal force, form a flowing film above the heavier seam then is conveyed by the shaking of the drum and continually washing water flow to the lower tilt inner end [[273], [274], [275]]. MGS was applied for the treatment of ultrafine coal as well as tailing pond deposits [276,277]. Statical optimization using response surface methodology based on Box-Behnken design (RSM-BBD) was used to analyze the effects of input parameters (i.e., drum inclination, drum rotation speed, and shaking frequency) on the output parameters (i.e., %yield, %ash, and %recovery) [[277], [278], [279]]. Similarly, empirical equations to estimate as a coal recovery were derived by Özgen et al. (2011) using a least square method via Minitab15 [274]. The result showed that drum inclination was determined as a major available on the reduction of ash content and other minor parameters are solid concentration, drum rotation speed, shaking amplitude, wash water rate, and feed rate [274]. Also, Özbakir et al. (2017) proposed a regression model with flow independent inputs (i.e., drum rotation speed, wash water rate, drum inclination, and solid concentration) and two dependent outputs (i.e., ash content and coal yield) variables [275]. The results showed that ash content and the yield were decreased when lower water flow rate, higher drum rotation speed, higher drum inclination, and solid concentration were used [275].

## 6 Critical discussion, future perspective, and conclusions

### 6.1 Critical discussion

There are many techniques employed in coal cleaning for preparation (e.g., crushing, screening, classification, microwave treatment, desliming, thickening, and filtration) and separation (i.e., gravity separation, flotation, oil agglomeration, magnetic separation, and electrical separation), which were introduced in this review. Among all the separation technologies, gravity-based separation is the most commonly used for coal cleaning because they are robust, easy to operate and maintain, requires low capital outlay and operational cost, provides high efficiency and capacity, and has minimal negative impacts to the environment.

Among of conventional gravity separation, the majority of studies in the last 10 years was about dense medium separator (DMS), particularly dense medium cyclone (DMC), while only a few works about jig, shaking table, and spirals were found. One reason for this trend is the decreasing quality of ROM coal, so fine grinding is required to achieve sufficient liberation. Because of this, the feed for the coal cleaning/processing plants are becoming finer and more difficult to handle with conventional coal cleaning technologies using water. Moreover, separation using dense media technologies are more effective compared with water-based methods and there are new and promising materials aside from magnetite that can be used as dense media. More recently, other new techniques like teeter bed separator (TBS) including Floatex density separator (FDS), CrossFlow separator, Allflux separator, Reflux separator, and inclined tapered diameter separation bed (ITDSB) were developed using the concept of sink-float separation similar to DMC but where autogenous medium from gangue minerals occurs when well-controlled water rising velocity is applied. These results show that the research trend is more focused on how to better and more effectively recover fine particles.

At present, coal cleaning using conventional gravity separators, including jig, dense medium cyclone, and spirals, is more widely used (about 80%) (Fig. 3; [67]); however, a lower number (75 papers) of research papers were published compared with that of dry type (96 papers) (Fig. S1). Most of the studies on conventional types, especially jigging and DMS, are about the improvement of separation efficiency using mathematical modeling, simulation of particle motion, process monitoring methods, and system controls. While for less common conventional methods like WOC and TBS, research is more focused on the development of the vessel as well as the modification of the separator.

In contrast, for dry-type gravity separation, many research papers were published, even though they are less popular. Most of the studies were focused on understanding the effects of various parameters because dry-type gravity separation for coal cleaning is still a new and emerging technology with lower separation efficiency compared with conventional wet-type gravity separation methods.

Lastly, the studies about EGS published the fewest papers (18). Most of the researchers studied the effects of various parameters, mechanisms, and separation optimizations. These techniques are still mostly studied at the laboratory level but are rarely used in real plants due to their low capacity, low separation efficiency, and high cost. However, the research about EGS is becoming more popular because it could improve the quality of coal into ultra-clean coal (UCC) via the reduction of impurities in fine particles that might increase the efficiency of the coal power plants and tend to be the low-carbon society (LCS).

This information showed that newer coal cleaning technology that suitable for fine particles are more focused on the effects of parameters and development of the equipment to improve the separation efficiency. While the conventional technology that more widely used in the real pants are more focused on numerical simulation and coal intelligently processing. Table 5 summarized the modeling and system control used in gravity separation for coal cleaning. It was found that 40% of papers on conventional gravity separation are about the modeling and system control while only about 10% for that of dry-type and EGS.

**Table 5:** Summary of modeling and system control used in gravity separation for coal cleaning.

Coal cleaning technology	Modeling	System control
Conventional gravity separation (75)	(15)	(15)
- Pulsation-type (17)	- CFD-DEM model (1) - Dynamic model using Matlab/Simulink (1) - Box-Behnken design (1)	- On-line measurement (1) - Feed control system (1) - 3D response surface (1) - Radiometric density (5)
- Flowing film-type (9)	N/A	- Automation and control system (1)
- Sink & float separation (27)	- CFD-DEM model (5) - Dynamic model (2) - Box-Behnken design (1) - Rosin-Rammler model (1) - Soft sensor model (1) - Solver and Mathcad (1)	- Smart sensor (1) - Model-data-based switching adaptive control (1) - Pump-storage system (1) - Medium density control (1) - Dual-loop control system (1) - Intelligent control (1)
- Others (22)	- Computational fluid dynamic and discrete element method (CFD-DEM) model (1)	N/A
Dry-type gravity separation (96)	(7)	(4)
- Fluidized separation w/dense medium (65)	- TFM-DEM hybrid model (3) - Magnetic particle surface attrition model (1) - Syamial-O’Brien drag model (1) - Disperses k-ϵ turbulence model (1)	- Density control (2) - Flow control (1)
- Fluidized separation w/o dense medium (12)	N/A	- Active pulsing air separation system (APSS) (1)
- Compound dry-type (19)	- The regression model (1)	N/A
Enhanced gravity separation (18)	(2)	N/A
- Pulsation-type (2)	N/A	N/A
- Fluidized bed-type (3)	- The regression model (1)	N/A
- Flowing (13)	- Box-Behnken design (1)	N/A
**Total (189)**	(24)	(19)

Note: The number in parenthesis shows the number of related papers.

N/A means not applicable.

### 6.2 Future perspective

As explained earlier, the recovery of fine particles is also becoming a big challenge in the processing of porphyry copper deposits by conventional mechanical flotation circuits [280]. This is because fine particles have very low momentum and tend to go around bubbles instead of colliding with them that decreases their probability of being recovered [86]. The idea from coal cleaning of agglomerating fine, hydrophobic coal using oil as a bridging liquid can be applied to mineral processing of porphyry copper deposits as well. This is because copper sulfide minerals like chalcopyrite are inherently hydrophobic like coal. Hornn and co-workers, for example, showed that by combining surfactants, oil and sufficient mixing before flotation, agglomeration of chalcopyrite could be induced that improved copper recovery [85,281,282]. Oil-agglomeration of fine particles can also be applied for the quantification and removal hydrophobic microplastics from contaminated soils, sediments, and water [283]. Lares et al. (2019), for example, used canola oil to improve the recovery and measurement of microplastics from wastewater treatment plant sewage sludge [284]. Finally, gravity-based recovery technologies for fine particles like DMC can be modified to separate metal-bearing fractions in electronic wastes as well as for the reprocessing of historic tailings before hydrometallurgical treatment. Jeon and co-workers, for example, reported that metals in electronic wastes (E-wastes) typically report to the fine fraction after crushing and grinding and can be recovered by gravity separation techniques [84,285]. Moreover, pretreatment of crushed E-wastes before leaching improved the extraction of gold dramatically [[286], [287], [288]].

Gravity-based separation technologies like float-and-sink and jigs can also be applied for the recycling of various kind of wastes (e.g., automobile [[289], [290], [291]], home appliances [292,293], small home appliances [294], mobile phones [285], copy machines [295], electrical wires [293,296], plastic containers [297], and mixed plastics [295,[298], [299], [300], [301]]. More recently, the advanced jig technology including reverse jig [297], hybrid jig [285,292,293,302], and reverse hybrid jig [296,303] were introduced to separate the mixed plastic waste that have similar SG as well as floating plastics.

In addition, dry-type gravity separation can also be modified for recycling applications. Na et al. (2020), for example, reported the separation of stainless steel and aluminum from discarded hard disk drives using zirconia balls as separation medium [304].

### 6.3 Conclusions

In this study, the works about gravity separation for coal cleaning in the last 10 years were reviewed. It was found that the studies have mainly contributed to the separation of fine coal as well as dry-coal separation, while conventional technologies are more focused on modeling to estimate and control the separation process. The UN-SDGs would likely catalyze more research efforts to limit the environmental impacts of coal throughout its life cycle including mining, logistics, processing/cleaning, and waste disposal/management. Although coal is frowned upon because of its contribution to CO_2_ emissions and climate change, this fossil fuel is crucial as a cheap and affordable source of energy for low-income and developing countries.

## Author contribution statement

Theerayut Phengsaart: Conceptualization, Methodology, Validation, Formal analysis, Writing – original draft, Writing – review & editing, Visualization, Project administration, Supervision. Palot Srichonphaisan: Validation, Formal analysis, Data curation, Writing – original draft, Writing – review & editing. Chinawich Kertbundit: Formal analysis, Writing – original draft. Natatsawas Soonthornwiphat: Formal analysis. Somthida Sinthugoot: Formal analysis, Writing – original draft. Nutthakarn Phumkokrux: Formal analysis, Writing – original draft. Onchanok Juntarasakul: Formal analysis, Visualization. Kreangkrai Maneeintr Writing – original draft, Supervision. Apisit Numprasanthai: Supervision. Ilhwan Park: Supervision. Carlito Baltazar Tabelin: Methodology, Writing – original draft, Writing – review & editing, Supervision. Naoki Hiroyoshi: Supervision. Mayumi Ito: Conceptualization, Writing – original draft, Writing – review & editing, Supervision.

All authors listed have significantly contributed to the development and the writing of this article.

## Funding statement

This research was supported by 10.13039/501100002873Chulalongkorn University [CU_FRB640001_01_21_4].

## Data availability statement

No data was used for the research described in the article.

## Declaration of competing interest

The authors declare that they have no known competing financial interests or personal relationships that could have appeared to influence the work reported in this paper.
