# Supplementary material for: Conventional and recent advances in gravity separation technologies for coal cleaning: A systematic and critical review
Source: Heliyon. 2023 Jan 21;9(2):e13083. doi: 10.1016/j.heliyon.2023.e13083 (PMC9922934; doi:10.1016/j.heliyon.2023.e13083)
Supplement: Multimedia component 1 [file mmc1.docx]

**Supplementary materials 1**


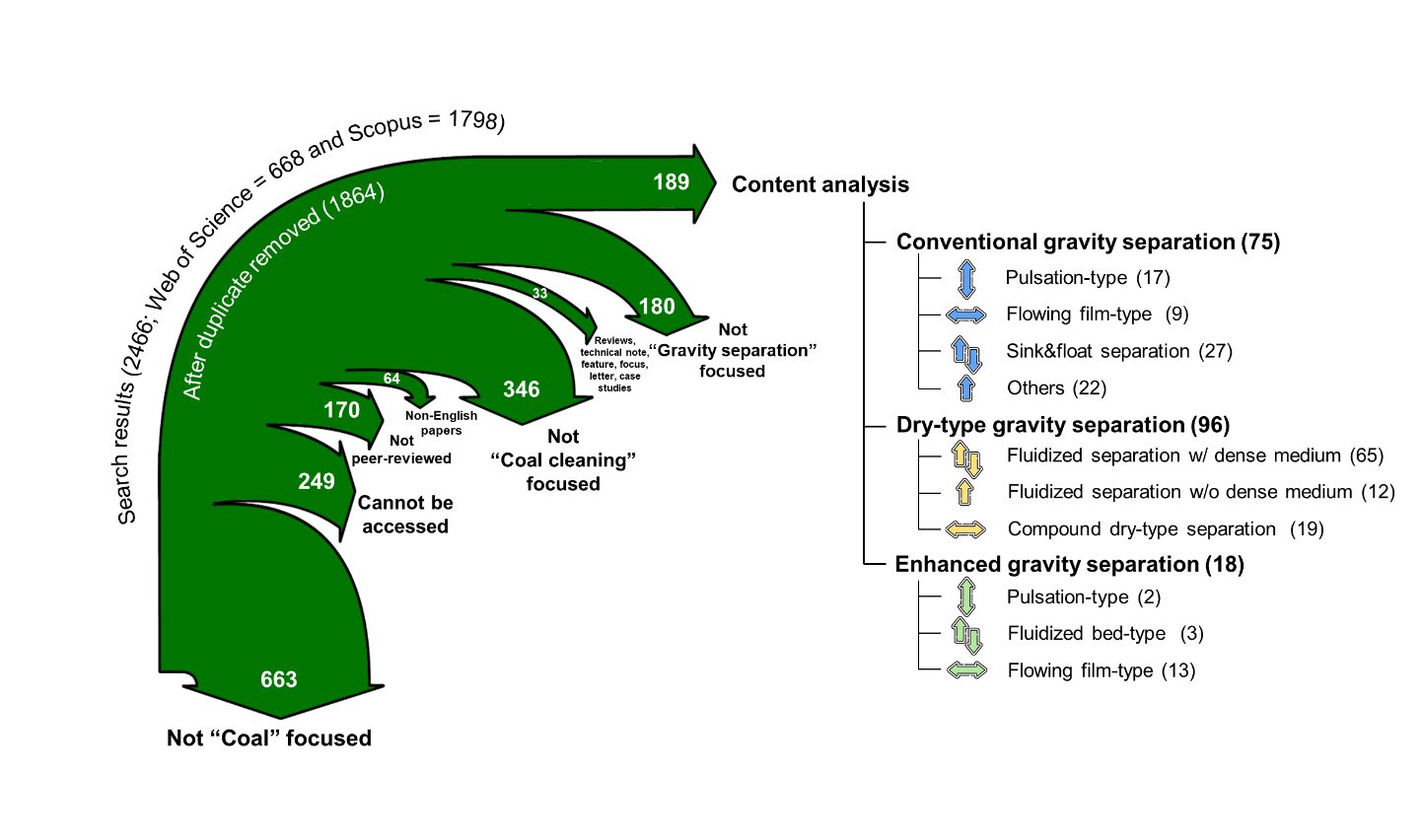


**Fig. S1** Study selection flow diagram

Fig. S1 shows the flow diagram of the study selection in this review paper. The literature was systematically reviewed to answer this question using the Preferred Reporting Items for Systematic Reviews and Meta-Analyses (PRISMA) guidelines to identify peer-reviewed journal publications that reported on “coal cleaning” and its synonyms, including “coal concentration”, “coal preparation”, “coal processing”, “coal beneficiation”, “coal separation” and “coal washing” together with technical keywords including “wet”, “dry”, “coarse”, “fine size”, “shape”, “density”, “settling velocity”, “gravity”, “centrifugal”, “jig”, “dense medium”, “dense media”, “cyclone”, “spiral”, “table”, and “fluidized bed”. Web of Science and Scopus were selected as databases for this systematic review, and the publication dates were limited to between 2010 and 2020 (i.e., 5 years before and after the ratification of UN-SDGs in 2015). The total search results are 2466 papers (from the Web of Science 668 papers and Scopus 1798 papers) and after removing duplicate papers, the remaining numbers are 1864 papers. In the screening step, titles, highlights, abstracts, and keywords were checked to remove those that did not focus on “coal”. The 663 papers were removed, and the other 1231 papers were moved to the next step. For the eligibility process, full-text articles were checked. The results showed that 249 papers cannot be accessed, 170 papers are not peer-reviewed, 64 papers are not written in English, 346 papers are not focused on “coal cleaning”, 33 papers are review papers, technical papers, feature, focus, letters, and case studies, and 180 papers are not focused on “gravity separation”. After the systematic selection, 189 papers remained and were used in this review. The selected papers are categorized into 3 main sections based on the contents; 75 papers go to conventional gravity separation (section 3), 96 papers go to dry-type gravity separation (section 4), and 18 papers go to enhance gravity separation (section 5).

**Table S1**

Recent studies of jig separation in coal cleaning.

| **Studies** | **References** |
| --- | --- |
| Effects of jig frequency of the separation efficiency of Alljig  (a laboratory side-pulsed Baum-type jig) | [94] |
| Effect of size feed distribution on the separation efficiency of Batac jig separation | [97] |
| Effects of changeable hydrodynamic parameters on the jig separation of fine coal | [103,104] |
| Statistical optimization of a laboratory mineral Denver jig for fine size high ash non-coking coal. | [98] |
| The on-line video analysis of feed particle size distribution | [106] |
| A simulation analysis of 4 control systems of feed (i.e., (i) volumetric, (ii) mass flow of material to a jig with additional measurement, (iii) bulk density of the feed, and (iv) ash content in the feed) | [110] |
| Numerical simulation to study the multiphase flow in an in-line pressure jig by using computational fluid dynamic (CFD) for water flow and discrete element method (DEM). | [110] |
| The monitoring of coal jig operation using radiometric density meter to simulate and stabilize the separation by understanding of coal/water density with the cycle of pulsation | [107–109,111] |
| A new simple dynamic model using Matlab/Simulink software to design and optimize control system of refuse discharge in a jig. | [105] |
| 3D response surface approach to estimate the quality of coal and separation efficiency of a laboratory model Denver jig | [95,96] |

**Table S2**

Summary of recent studies on DMC in coal cleaning.

| **Studies** | **Description** | **References** |
| --- | --- | --- |
| **Vessel** | Three-products DMC | [125,126] |
|  | Tri-Flo separator | [127,128] |
| **Medium** | Magnetite, clay, and fine coal mixtures with different ratio | [130] |
|  | Silica-based media (i.e., silica sand, silica fume, and fine silicon) | [129] |
| **Modeling** | Three-dimension computational fluid dynamic (3-D CFD) simulation | [131] |
|  | Computational fluid dynamic and discrete element method (CFD-DEM) model | [133] |
|  | PC-based mathematic model based on CFD-DEM | [134] |
|  | CFD-DEM with Johnson’s SB function | [135] |
|  | Computational fluid dynamic (CFD) using discrete phase model and Algebraic Slip mixture multiphase model | [132] |
|  | Dynamic model | [136] |
|  | Dynamic and steady-state model | [137] |
|  | Response surface methodology based on Box Behnken Design (RSM-BBD) and artificial neural network (ANN) | [279] |
|  | Rosin-Rammler model | [138] |
|  | Soft sensor model | [120] |
|  | Solver (an optimization routine available in Excel) | [122] |
|  | Solver and Mathcad | [123] |
| **Systems control** | Smart sensor | [130] |
|  | Model-data-based switching adaptive control | [139] |
|  | Pump-storage system | [195] |
|  | Medium density control | [141] |
|  | Dual-loop control system | [142] |
|  | Intelligent control including electronic belt scale, ash scanners, liquid level meters, densimeter, magnetite content meter, and pressure gage | [143] |

**Table S3**

Important parameters of vibrated fluidized bed separator in coal cleaning.

| **Parameters** | **Details** | **References** |
| --- | --- | --- |
| **Feed rate** | Increasing feed rate decreases separation performance | [236] |
| **Superficial air velocity** | Superficial air velocity is the most important parameter that affects the separation of fine coal. Smaller value of superficial air velocity could achieve better separation performance. | [231–235] |
| **Amplitude** | Decreasing amplitude could increase the separation efficiency. | [230,231,233,234] |
| **Frequency** | Decreasing frequency could increase the separation efficiency. | [230,231,233–235] |
| **Initial bed height** | Initial bed height is not the main parameter affecting the separation efficiency. | [231,234] |
| **Separating time** | Suitable separation time should be controlled since the mixing could occur with overmuch time. | [230] |
| **Bubble size** | When the bubble size is too small, there is not enough space for particle settling while when bubble is too big, the rising velocity of bubble become too fast to provide enough time for particle settling so, the optimal control of bubble size is needed. | [231] |

**Table S4**

Important parameters of compound dry separation in coal cleaning.

| **Parameters** | **Description** | **References** |
| --- | --- | --- |
| **Particle size** | The finer particles can cause positive result in combustible content separation. | [250–253] |
| **Feed rate** | Lower feed rate could positively affect the separation. | [246] |
| **Air flow rate** | Higher air flow rate could decrease the ash content in clean coal product since sufficient air velocity is required to remove heavy inorganic impurities. | [249,254,255] |
| **Table transverse inclination** | Table transverse inclination can cause forward movement of high ash particles to discharge point. Increasing table transverse inclination could increase the combustible content of clean coal (by reducing ash content) however, clean coal yield also decreases. | [249,256] |
| **Table longitudinal inclination** | Decreasing longitudinal angle can also affect more ash particles to move to the end of reject stream which can raise combustible content of clean coal. | [246,256] |
| **Table vibration frequency** | Increasing vibration frequency influences ash particles moving forward to join with reject seam resulting in higher separation efficiency. | [246] |
| **Riffle height** | Increasing riffle height has positive effects to the separation. | [246] |
| **Partition plate height** | Increasing partition plate height can significantly block ash content in clean coal stream followed by length and angle. | [248,257–259] |
